# Effects of salt stress on plant and rhizosphere bacterial communities, interaction patterns, and functions

**DOI:** 10.3389/fpls.2024.1516336

**Published:** 2025-01-09

**Authors:** Maoxing Fu, Liying Liu, Bingzhe Fu, Meiling Hou, Yanzi Xiao, Yinghao Liu, Duowen Sa, Qiang Lu

**Affiliations:** ^1^ Key Laboratory of Innovation of Forage Efficient Production Model, Ministry of Agriculture and Rural Affairs, Yinchuan, Ningxia Hui Autonomous Region, China; ^2^ College of Forestry and Prataculture, Ningxia University, Yinchuan, China; ^3^ Inner Mongolia Autonomous Region Forestry Scientific Research Institute, Hohhot, China; ^4^ College of Life Science, Baicheng Normal University, Baicheng, China; ^5^ Agricultural College, Hulun Buir College, Hailar, China; ^6^ Grassland Research Institute, Chinese Academy of Agricultural Sciences, Hohhot, China

**Keywords:** soil salinity, microbial community, microbial metabolic function, chemical composition, amino acids

## Abstract

**Introduction:**

Salt stress significantly affects plant growth, and Na^+^ has gained attention for its potential to enhance plant adaptability to saline conditions. However, the interactions between Na^+^, plants, and rhizosphere bacterial communities remain unclear, hindering a deeper understanding of how Na^+^ contributes to plant resilience under salt stress.

**Methods:**

This study aimed to investigate the mechanisms through which Na^+^ promotes alfalfa's adaptation to salt stress by modifying rhizosphere bacterial communities. We examined the metabolic activity and community composition of both plant and rhizosphere bacteria under Na^+^ treatment.

**Results and discussion:**

Our results revealed significant changes in the metabolism and community composition of both plant and rhizosphere bacteria following Na^+^ addition. Na^+^ not only promoted the growth of rhizosphere bacteria but also induced shifts in the plant-associated bacterial community, increasing the abundance of bacterial species linked to alfalfa's resistance to salt stress. Furthermore, the chemical characteristics of alfalfa were strongly correlated with the composition and network complexity of both plant and rhizosphere bacterial communities. These interactions suggest that Na^+^ plays a crucial role in enhancing alfalfa’s adaptability to salt stress by fostering beneficial bacterial communities in the rhizosphere. This finding highlights the potential of leveraging Na^+^ interactions with plant-microbe systems to improve crop resilience and productivity in saline agricultural environments.

## Introduction

Worldwide, salinity stress is an increasingly severe soil degradation issue that significantly affects plant growth and development (Gong et al., 2023). Each year, around 10 million hectares of irrigated farmland are abandoned, with approximately 50% affected by salt stress (Khasanov et al., 2023). This problem is expected to worsen in the future. Various salt ions in the soil, including sodium (Na^+^), bicarbonate (HCO^3−^), sulfate (SO_4_^2−^), potassium (K^+^), chloride (Cl^−^), calcium (Ca^2+^), and carbonate (CO^2−^), dissolve in water, causing salinization (Hu et al., 2022). Sodium chloride (NaCl) is the main component among these salts, and Na^+^ is toxic to plants, inhibiting their growth at high concentrations. The accumulation of Na^+^ in plant tissues inhibits photosynthesis and enhances the formation of reactive oxygen species (ROS) (Fu et al., 2023). These ROS have many adverse effects on plants, such as accelerating toxic reactions, leading to DNA mutations, protein degradation, and membrane damage (Sharmin et al., 2022).

Although plant salt adaptation is often attributed to genetic differentiation, the microbiota has recently been recognized as a key factor in plant stress tolerance. Within plant-associated microbiota, plant growth-promoting rhizobacteria (PGPR) have emerged as crucial players in enhancing plant performance under salinity conditions. This enhancement is achieved through various beneficial processes, including mediating ion homeostasis, producing phytohormones, promoting osmolyte accumulation, enhancing antioxidant activity, adapting metabolic mechanisms, and facilitating nutrient absorption (Wang et al., 2023). The activity and diversity of PGPR are influenced by root exudates (Feng et al., 2023; Sasse et al., 2018), which can be altered by various abiotic stresses such as nutrient deficiency, drought, and salinity (Schmitz et al., 2022). PGPR employs various mechanisms to promote plant growth, such as stimulating root and shoot growth by producing phytohormones like auxins and cytokinins (Ajilogba et al., 2022).

Inoculation of plants with inter-root bacteria (ST-PGPR) can produce antioxidant enzymes to mitigate oxidative stress. In saline Tunisian soils, the salt-tolerant plant *Sulla carnosa* exhibited enhanced growth and stress tolerance after inoculation with strains of *Pseudomonas* sp. and *Bacillus* sp (Hidri et al., 2016). The induction of antioxidant enzymes by ST-PGPR is a key mechanism behind their beneficial effects on plant performance under salinity stress. Khoso et al. (2023) and Ansari et al. (2019) have demonstrated that ST-PGPR strains produce elevated levels of antioxidant enzymes, including peroxidase (POD), superoxide dismutase (SOD), catalase (CAT), nitrate reductase (NR), and glutathione reductase (GR), under saline stress conditions. This enzymatic production plays a critical role in mitigating oxidative stress and enhancing plant tolerance to salt stress. El-Esawi et al. (2019) reported that inoculating chickpea plants with *Azospirillum lipoferum* FK1 enhanced nutrient uptake, increased antioxidant gene expression, improved antioxidant enzyme activity, and promoted the accumulation of non-enzymatic metabolites, thereby supporting overall plant growth and development. Based on the above findings, it is evident that ST-PGPR plays a crucial role in enhancing plant stress tolerance by regulating various physiological and biochemical processes. These processes include modulating stress-related genes, accumulating osmolytes, and regulating both enzymatic and non-enzymatic antioxidant systems.

The activity of plant growth-promoting rhizobacteria (PGPR) is influenced by the level of salt stress (Wang et al., 2022). Liu et al. (2022) also observed that enzyme types and amino acid composition change under various abiotic stress conditions. The multifaceted functions of nonspecific root exudates, along with the potential for synergistic interactions in the plant-soil system, could address the bottlenecks in efficient nutrient utilization; however, these mechanisms remain poorly understood. Based on these observations, it is hypothesized that plants under salt stress conditions recruit specific plant growth-promoting rhizobacteria (PGPR) to enhance their salt stress tolerance. These beneficial microorganisms are believed to positively influence various physiological and biochemical processes in plants, such as osmolyte accumulation and the modulation of enzymatic and non-enzymatic antioxidant systems.

To test the hypotheses, we focused on alfalfa (*Medicago sativa* L.), a species known for its wide variation in salt stress resistance. This study examined the physiological responses of cultivated alfalfa varieties to salt treatments (specifically sodium chloride [NaCl]) and evaluated the functional redundancy of rhizospheric microorganisms in enhancing plant adaptability to salt stress. Our objectives were to investigate: (i) whether plants can selectively recruit specific PGPB to enhance salt stress tolerance, (ii) whether salt stress alters amino acid composition and enzyme activity to facilitate better adaptation, and (iii) whether functional redundancy exists among PGPB in alleviating salt stress. By gaining a deeper understanding of the complex mechanisms of plant–PGPB interactions under salt stress, we aim to unlock the potential of these microorganisms for sustainable agriculture.

## Materials and methods

### Plant materials and salt stress treatment

The alfalfa variety used in this experiment was Zhongmu 3, provided by the Institute of Animal Science and Veterinary Medicine, Chinese Academy of Agricultural Sciences, Beijing. To sterilize the seeds, they were first treated with 75% ethanol for 30 s, followed by five washes with distilled water. The seeds were then disinfected with 4% sodium hypochlorite for 3 min and rinsed 10 times with distilled water until no odor remained. The surface moisture was absorbed using filter paper.

The seeds were subsequently sown in seedling trays (50 holes per tray) and placed in a growth chamber for germination. Once the seedlings developed two to three true leaves and exhibited uniform, robust growth, they were transferred to soils with different salt stress levels for further cultivation. The experimental conditions included four salt stress treatments: nonsalt stress (CK), light salt stress (SL), moderate salt stress (SZ), and severe salt stress (SH). The NaCl concentrations for the CK, SL, SZ, and SH treatments were < 1‰ (0 mmol/L), 1‰–2‰ (20 mmol/L), 2‰–3‰ (40 mmol/L), and 3‰–4‰ (60 mmol/L), respectively. Each treatment was replicated three times.

The plants were allowed to grow for a total of 7 weeks, reaching the initial flowering stage, at which point measurements and samples were taken for analysis. The physical properties of the soil are provided in **Supplementary Table S1**. The soil used for this experiment was sourced from a field at Ningxia University, and salt stress was applied by adding NaCl to the soil to achieve the desired salt concentrations.

### Chemical composition of alfalfa

Aboveground biomass (AGB) was estimated by harvesting the aboveground portions of the plants at different growth stages. The dry matter (DM) of fresh alfalfa and silage was determined by oven drying at 65°C for 72 h. Neutral detergent fiber (NDF) and acid detergent fiber (ADF) were measured following the procedures described by Van Soest et al. (1991). Starch and water-soluble carbohydrate (WSC) content were determined using colorimetry after reaction with anthrone reagent (Yemm and Willis, 2021). Nonstructural carbohydrates (NSC) were the sum of WSC and starch content. Crude protein (CP = total *N* × 6.25) was determined using a Kjeldahl apparatus (Gerhart Vapodest 50s, Germany), following the method of Kjeldahl (1883) with recent adaptations in modern Kjeldahl methods (Marcó et al., 2002). Soluble protein (SP) was measured using the trichloroacetic acid method (Cunniff, 1997), with updates to protein quantification procedures (Li et al., 2024). Na^+^ and K^+^ ion concentrations in alfalfa were measured relative to standard solutions using a model 425 flame photometer (Sherwood Scientific Ltd, UK), as described by Jankowski and Freiser (1961). Amino acid content was determined using an automatic amino acid analyzer (Hitachi L-8900 Amino Acid Analyzer), with results quantified by peak area using the external standard method (Spackman et al., 1958).

### Bacterial community analysis

Total genomic DNA was extracted from compost samples using the E.Z.N.A.^®^ Plant DNA Kit (Omega Bio-Tek, Norcross, GA, USA) following the manufacturer’s recommendations. The V3–V4 variable regions of bacterial 16S rRNA were amplified using primers 338F (5′-ACTCCTACGGGAGGCAGCAG-3′) and 806R (5′-GGACTACHVGGGTWTCTAAT-3′). Operational taxonomic units (OTUs) were clustered with a 97% similarity cutoff using UPARSE. The taxonomy of each 16S rRNA gene sequence was analyzed using the RDP Classifier algorithm against the Silva (SSU123) 16S rRNA database, with a confidence threshold of 70%. Functional prediction was performed using bioinformatics tools such as DIAMOND, which aligns the sequences with KEGG metabolic pathways, and HUMAnN2 for microbial functional pathway analysis. This approach helped identify the functional pathways associated with the microbial community’s ability to process lignocellulose and other substrates.

### Statistical analysis

Variations in physicochemical parameters were analyzed using SAS (version 9.3, SAS Institute Inc., Cary, NC, USA). All measurements were conducted in triplicate. Core bacterial community network analysis was performed using Gephi (version 0.10.1). Structural equation modeling (SEM) to evaluate differences among treatments was conducted using IBM SPSS Statistics 27.0.

## Results

### Effect of agronomic traits on alfalfa response under salt stress

**Figure 1:**
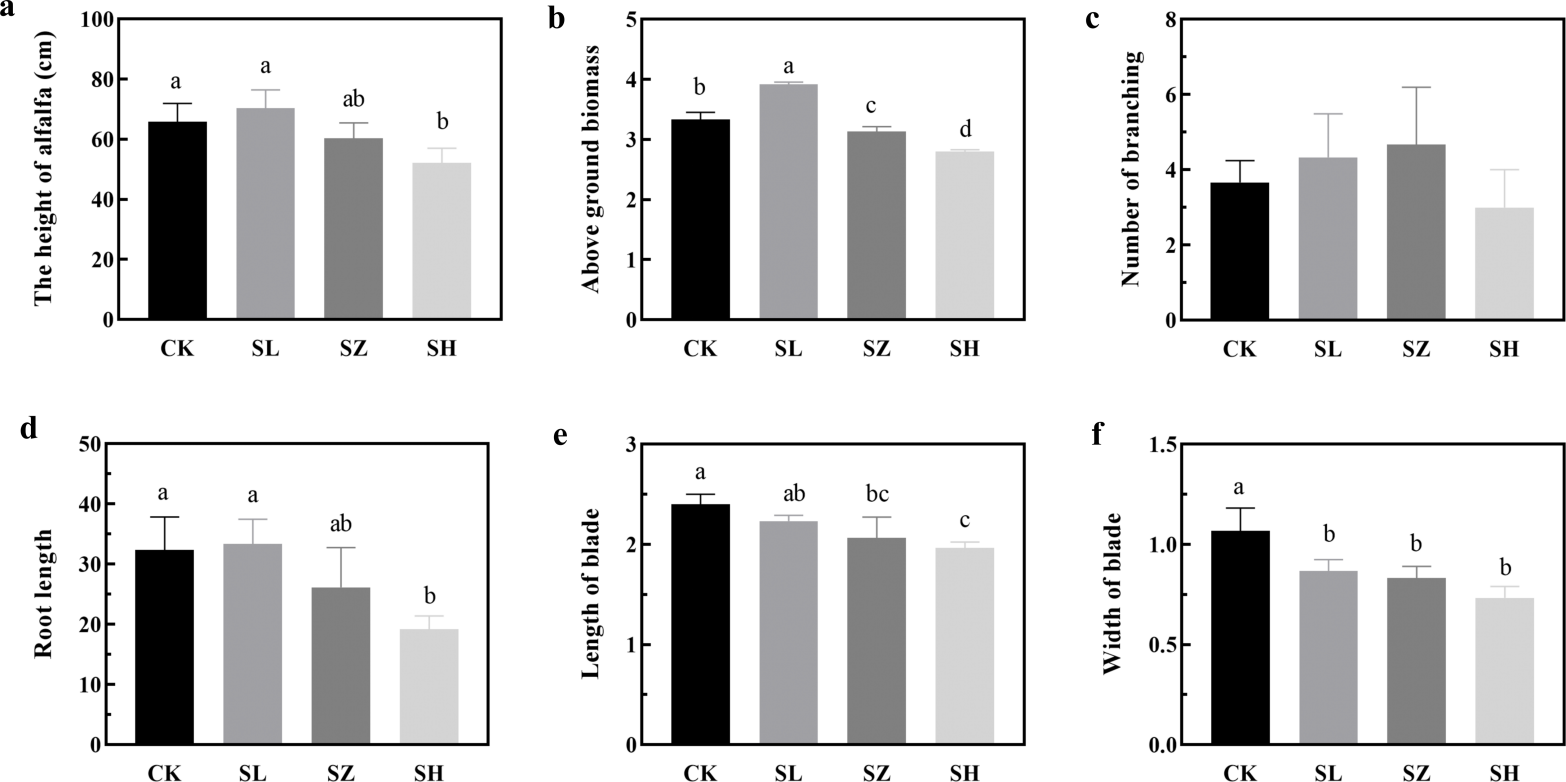
Effect of salt stress on agronomic traits of alfalfa: **(A)** alfalfa height; **(B)** aboveground biomass; **(C)** number of branches; **(D)** root length; **(E)** blade length; and **(F)** blade width. CK, without salt stress; SL, light salt stress; SZ, middle salt stress; SH, severe salt stress. Columns with different letters indicate significant differences (*p* < 0.05).

To highlight the importance of salt stress on plant fitness, we established an assay to study alfalfa growth under conditions mimicking its native environment. Salt stress significantly impacts the agronomic traits of alfalfa under indoor conditions (**Figure 1**). Alfalfa height was significantly greater in the CK and SL treatments compared to SH (*p* < 0.05), ranging from 50 to 80 cm. Blade length in CK was significantly greater than in SZ and SH (*p* < 0.05), while blade width in CK was significantly greater than in the other treatments (*p* < 0.05). Additionally, the SL treatment exhibited significantly higher aboveground biomass than the other three treatments (*p* < 0.05), whereas SH showed the lowest, ranging from 2 to 4 kg/m². Root length in CK, SL, and SZ was significantly greater than in SH (*p* < 0.05), ranging from 15 to 40 cm.

### Salt stress-induced changes in chemical properties of alfalfa

**Figure 2:**
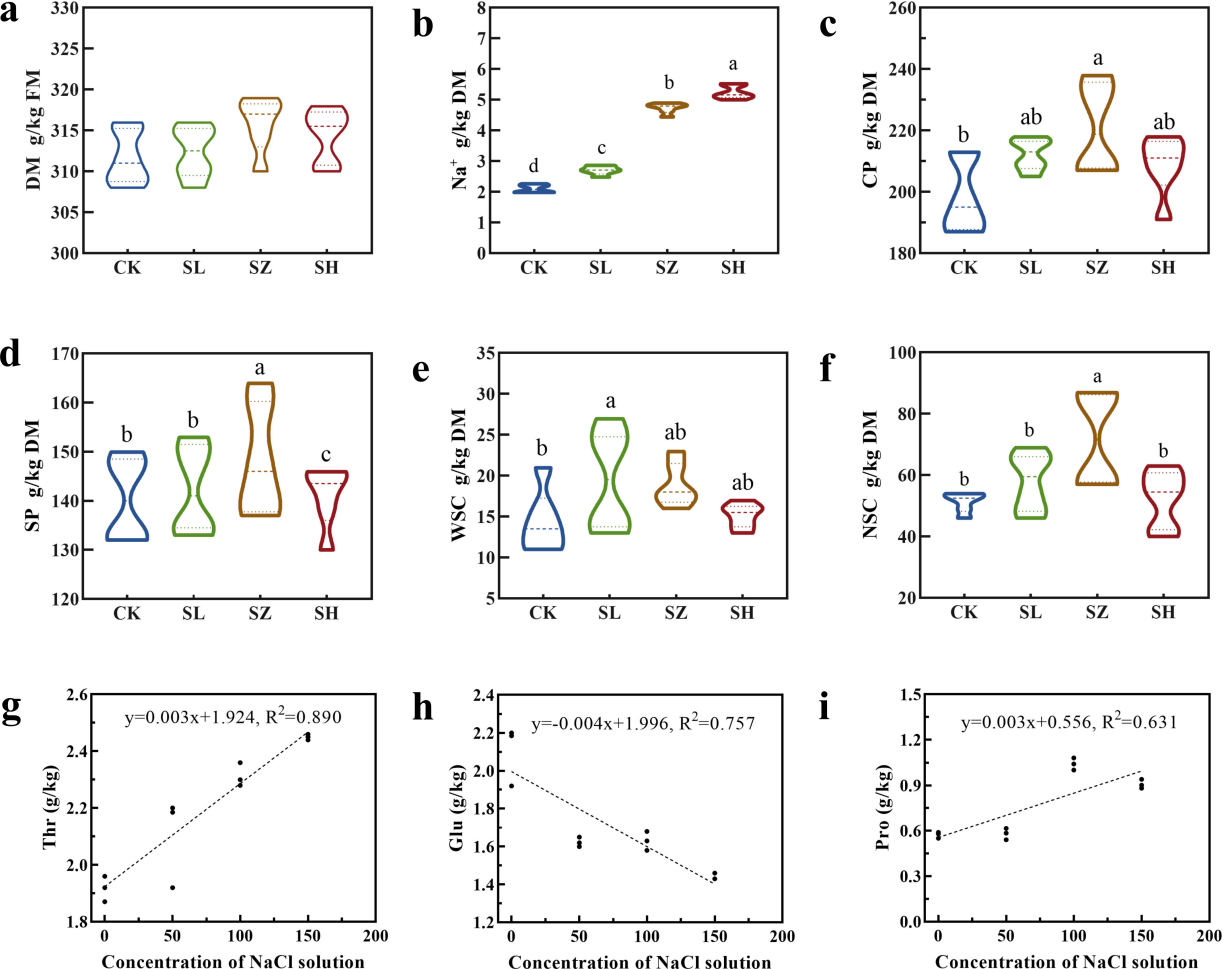
Effect of salt stress on chemical compositions and amino acids in alfalfa: **(A)** dry matter (DM), **(B)** Na^+^ content, **(C)** crude protein (CP), **(D)** soluble protein (SP), **(E)** water-soluble carbohydrate (WSC), **(F)** nonstructural carbohydrate (NSC), **(G)** threonine (Thr) content, **(H)** glutamic acid (Glu) content, and **(I)** proline (Pro) content. CK, without salt stress; SL, light salt stress; SZ, middle salt stress; SH, severe salt stress. Columns with different letters indicate significant differences (*p* < 0.05).

We investigated whether salt stress could induce changes in the chemical and amino acid characteristics of alfalfa (**Figures 2A–F**). The DM content in SL, SZ, and SH was significantly higher than in CK (*p* < 0.05). In the SZ treatment, the DM content ranged from 310 to 320 g/kg. Our results show that salt stress did not inhibit alfalfa DM content; instead, SL, SZ, and SH treatments significantly promoted it. The Na^+^ content in SH was significantly higher than in the other three treatments (*p* < 0.05), while SZ and SL showed significantly higher Na^+^ content than CK (*p* < 0.05), ranging from 2 to 6 g/kg. The results showed that SL, SZ, and SH treatments promoted Na^+^ content. Additionally, CK had significantly lower CP content than the other treatments (*p* < 0.05). In the SZ treatment, the CP content ranged from 200 to 240 g/kg. The SP content in SZ was significantly higher than in the other three treatments (*p* < 0.05), with values ranging from 130 to 170 g/kg. The WSC content in SL was significantly higher than in the other three treatments (*p* < 0.05), while SZ and SH showed significantly higher WSC content than CK (*p* < 0.05), ranging from 10 to 30 g/kg. The NSC content in SZ was significantly higher than in the other three treatments (*p* < 0.05), ranging from 40 to 100 g/kg.

To explore the impact of salt stress on alfalfa amino acids, these graphs (**Figures 2G–I**) illustrate the variations in amino acid content in response to increasing salt concentrations, providing insights into the plant’s biochemical responses. The relationship between NaCl concentration and the amino acids Thr (*y* = 0.003*x* + 1.924, *R* ^2^ = 0.890) and Pro (*y* = 0.003*x* + 0.556, *R* ^2^ = 0.631) in plant tissues suggests an increase in Thr with rising NaCl levels, indicating an adaptive response to salt stress. Although the correlation for Pro is not as strong as for Thr, it still indicates an increase in Pro content at higher salt concentrations, aligning with common plant stress responses where Pro accumulates as part of an osmoprotective strategy. Conversely, Glu content decreased with increasing salt concentration (*y* = − 0.004*x* + 1.996, *R* ^2^ = 0.757). This suggests that Glu may be depleted or reallocated under stress conditions, unlike Thr and Pro, which exhibited different trends.

### Salt stress-induced changes in endophytes of alfalfa

**Figure 3:**
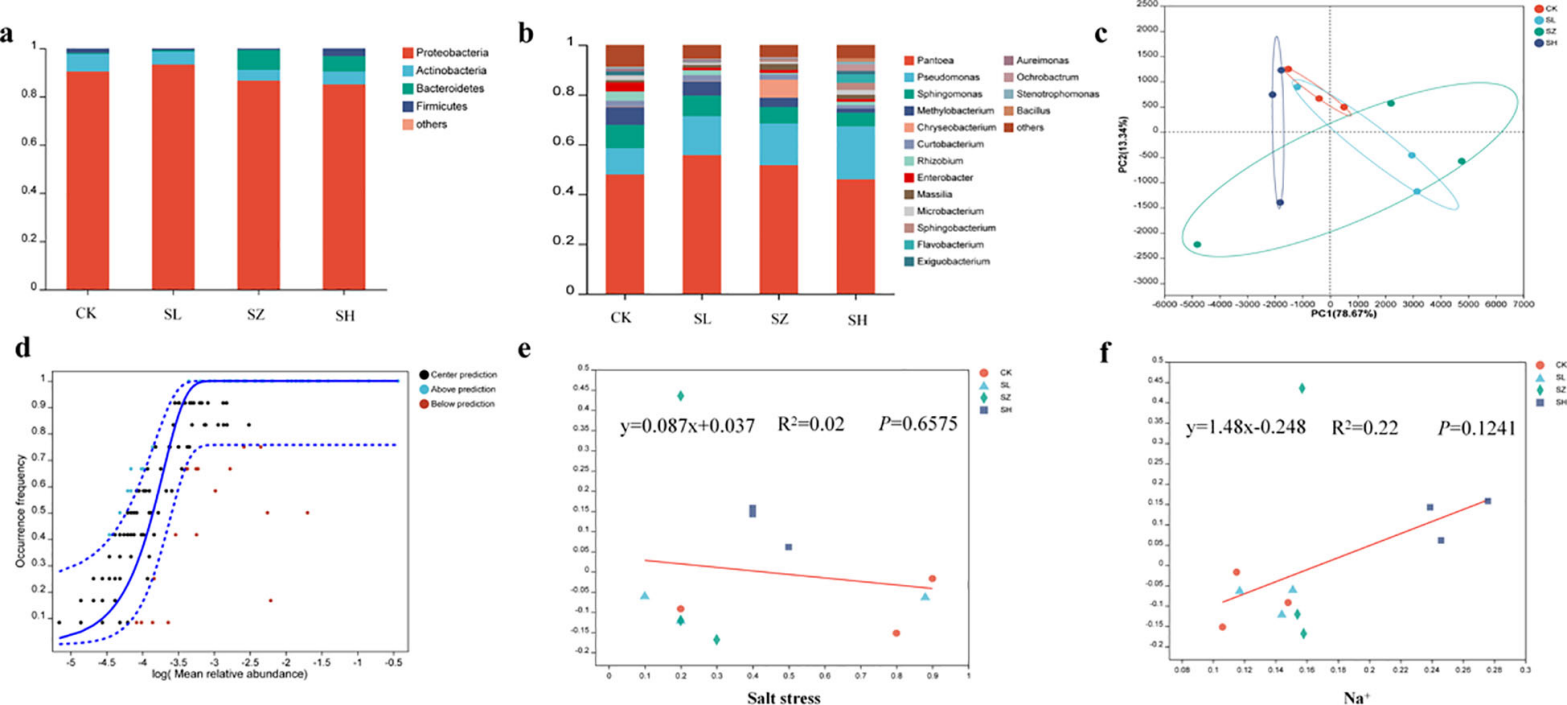
Diversity of microbial community structure in alfalfa bacteria: **(A)** microbial composition of the phylum, **(B)** microbial composition of the genus, **(C)** plot of principal component analysis of plant bacterial community, **(D)** neutral community modeling of plant bacterial community, **(E)** regression analysis of soil salt stress and plant bacterial communities, and **(F)** regression analysis of plant Na^+^ and bacterial communities. CK, without salt stress; SL, light salt stress; SZ, middle salt stress; SH, severe salt stress.

Salt stress can alter the composition of plant microbiota (Chinnaswamy et al., 2018), as demonstrated in previous studies. Salinity-induced changes in the bacterial community structure of alfalfa were characterized through sequencing analysis, **Figure 3** depicts the composition of the bacterial community in alfalfa subjected to salt stress. The dominant bacterial families in alfalfa under different salt treatments (CK, SL, SZ, and SH) were *Proteobacteria*, *Actinobacteria*, *Bacteroidetes*, and *Firmicutes*, with their relative abundances varying across the treatments. The abundance of *Proteobacteria* was observed to increase in the SL treatment compared to the control (CK), but it decreased again in the SZ and SH treatments. This variation is across the different salt treatments indicates how varying levels of salt stress influence the abundance of *Proteobacteria* in alfalfa. The abundance of *Actinobacteria* was consistently higher in the CK group than in the SL-, SZ-, and SH-treated groups, regardless of salt concentration. The abundance of *Bacteroidetes* followed the trend: CK < SL < SH < SZ.

At the genus level, salt stress induced significant shifts in the bacterial community composition (**Figure 3B**). The dominant genera across all treatments were *Pantoea*, *Pseudomonas*, and *Sphingomonas*, although their abundances varied. The abundance of *Pantoea* initially increased but subsequently decreased across the treatments, with the lowest abundance in the CK group and the highest abundance in the SL group. The abundance of *Pseudomonas* gradually increased from the CK to the SH treatment group, reaching its highest level in the SH group. In contrast to *Pseudomonas*, the abundances of *Sphingomonas*, *Methylobacterium*, *Enterobacter*, and *Rhizobium* were significantly lower in the SL, SZ, and SH treatment groups compared to the CK group. The abundance of *Chryseobacterium* was significantly higher in the SZ treatment, while the abundance of *Flavobacterium* was significantly higher in the SH treatment.

PCoA analysis revealed distinct clustering of microbial communities across the four treatments (**Figure 3C**). The CK and SL treatments formed overlapping clusters, suggesting similar microbial community structures. The SH treatment clustered distinctly to the left, while the SZ treatment was more distant, indicating greater dissimilarity in community structure compared to the other treatments. Neutral community modeling (**Figure 3D**) illustrated the impact of salt stress on alfalfa microbial communities. Salt concentration had a significant impact on microbial communities, as revealed by the NCM model, which successfully captured the relationships between Na^+^ levels and microbial composition. Model fit (*R* ^2^) assessed the coherence of microbial communities. Beta diversity increased in the order SH > CK > SL > SZ as salt stress increased, as indicated by the regression equation *y* = 0.087*x* + 0.037 (**Figure 3E**). Salt stress had a significant positive correlation with beta diversity of alfalfa microbial communities, indicating its influence on their structure. Beta diversity was highest in the SZ treatment, as indicated by the regression equation *y* = 1.48*x* − 0.248 (**Figure 3F**).

### Salt stress induces the recruitment of specific bacterial communities in the rhizosphere

**Figure 4:**
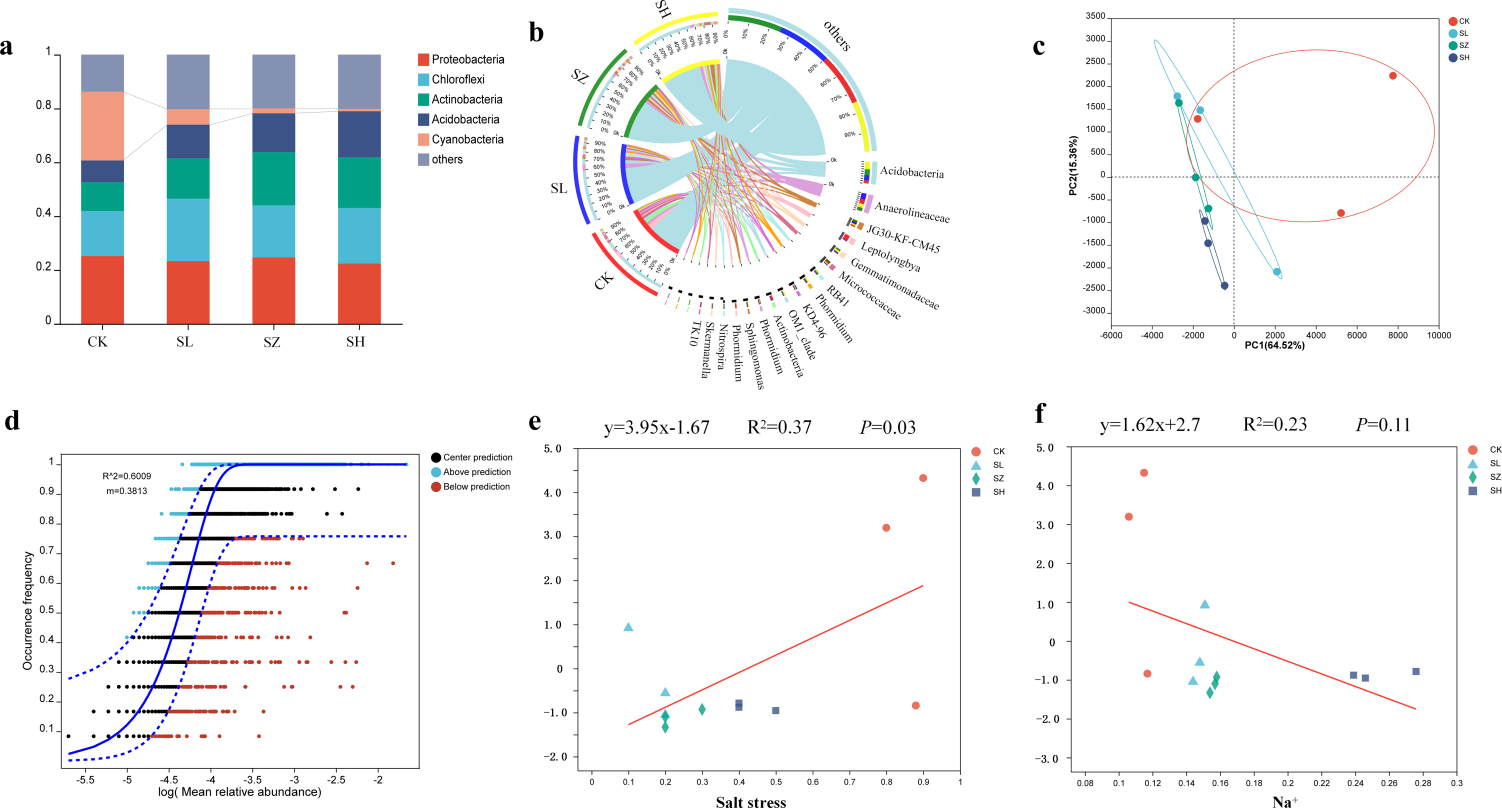
Diversity of microbial community structure in alfalfa rhizosphere bacteria: **(A)** microbial composition of the phylum, **(B)** microbial composition of the genus, **(C)** plot of principal component analysis of rhizosphere bacterial community, **(D)** neutral community modeling of the rhizosphere bacterial community, **(E)** regression analysis of soil salt stress and rhizosphere bacterial communities, and **(F)** regression analysis of plant Na^+^ and rhizosphere bacterial communities. CK, without salt stress; SL, light salt stress; SZ, middle salt stress; SH, severe salt stress.

To investigate the dynamic succession of rhizosphere bacteria in alfalfa under salt stress, we constructed and sequenced 16S rRNA amplicon libraries using Illumina technology. The rhizosphere bacterial communities of alfalfa exhibited significant differences at the phylum level across the CK, SL, SZ, and SH treatments. Five phyla were dominant in the rhizosphere bacterial communities of alfalfa (**Figure 4A**). The abundances of *Actinobacteria* and *Acidobacteria* gradually increased across the CK, SL, SZ, and SH treatment groups. In contrast, the abundance of cyanobacteria in the CK, SL, SZ, and SH treatment groups gradually decreased. Bacterial diversity varied among the different treatments (**Figure 4B**). Salt stress inhibited the growth of *Leptolyngbya* in the SL, SZ, and SH treatments, while the growth of *Actinobacteria* was suppressed in the CK and SL treatments. The abundance of RB41 increased, favoring its growth in the CK, SL, and SH treatments. The growth of Anaerolineaceae was also promoted in the CK, SL, and SH treatments.

PCoA ordination revealed distinct differences in rhizosphere bacterial communities among the four treatments (**Figure 4C**). The SZ and SH treatments clustered to the left, while the CK treatment was more distant, indicating that the rhizosphere bacterial communities of the other three treatments differed significantly from the CK treatment. The rhizosphere community structure of the SZ, SL, and SH treatments differed significantly from the CK treatment. NCM analysis revealed changes in the rhizosphere bacterial communities of alfalfa under salt stress (**Figure 4D**). Salt stress significantly impacted the rhizosphere bacterial communities of alfalfa, providing insights into their dynamics. The NCM model effectively captured the relationships between Na^+^ salt concentration and the plant rhizosphere bacteria. Model goodness of fit (*R*²) assessed the degree of community aggregation. The regression equation for the relationship between salt stress and alfalfa rhizosphere bacterial community structure was *y* = 3.95*x* − 1.67 (**Figure 4E**). Beta diversity of the alfalfa rhizosphere bacterial community decreased in the order CK > SL > SH > SZ as salt stress increased. Salt stress had a significant positive correlation with the beta diversity of the alfalfa rhizosphere bacterial community, indicating its impact on community structure. The regression equation for the relationship between Na^+^ and alfalfa rhizosphere bacterial community structure was *y* = 1.62*x* + 2.7 (**Figure 4F**). Beta diversity of the alfalfa rhizosphere bacterial community decreased in the order CK > SL > SH > SZ. Salt stress and Na^+^ had significant effects on the structure and diversity of the alfalfa rhizosphere bacterial community. These findings shed light on how salt stress alters microbial community dynamics, which is important for understanding plant–microbe interactions under stress conditions.

### Effects of salt stress on the metabolic functions of plant and rhizosphere bacteria

**Figure 5:**
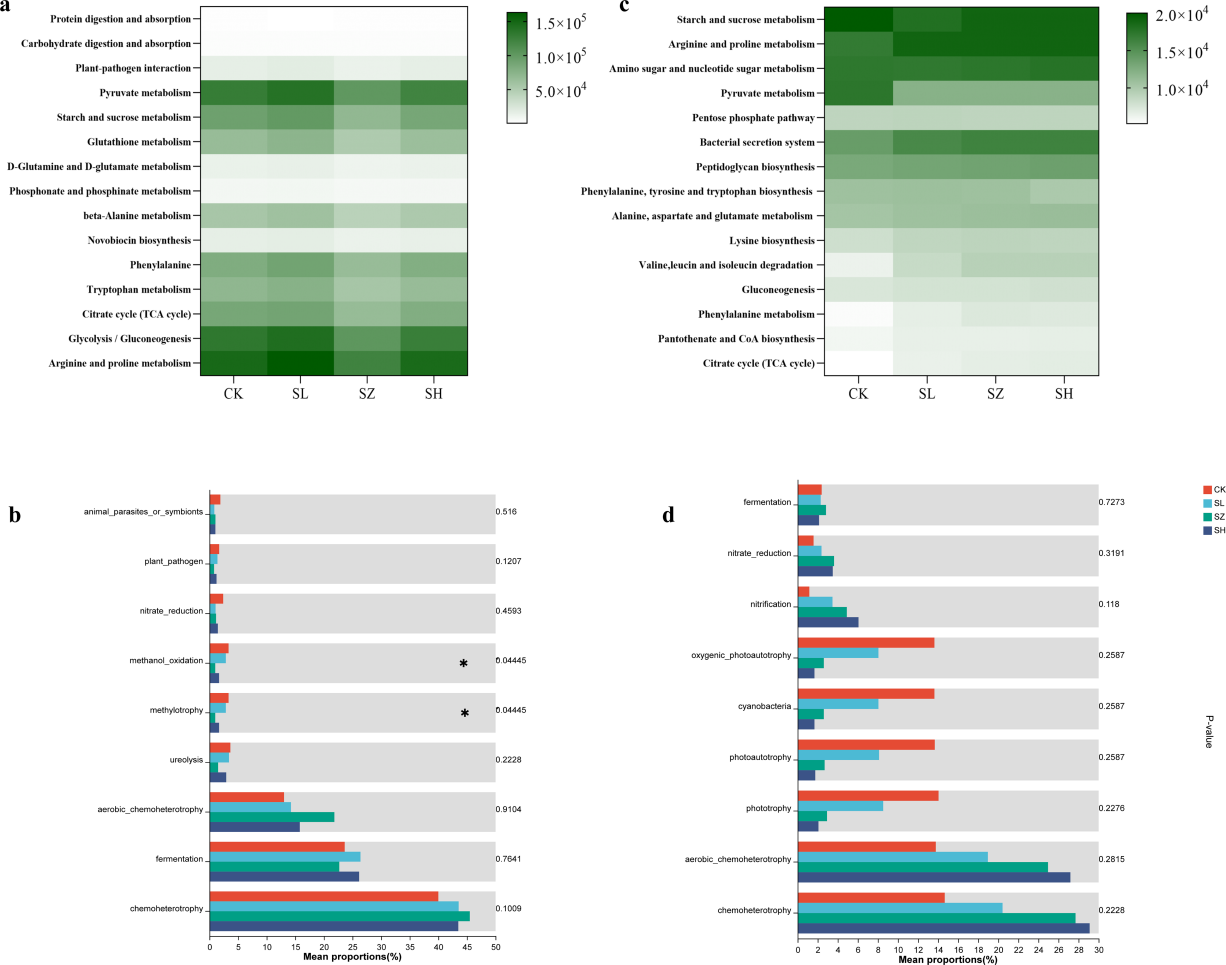
Effects of soil salt stress on metabolic differences in plants and rhizosphere bacterial communities: KEGG pathways in **(A)** plant and **(B)** rhizosphere bacterial communities; differential analysis of the metabolic functions of **(C)** plants and **(D)** rhizosphere. CK, without salt stress; SL, light salt stress; SZ, middle salt stress; SH, severe salt stress.

The salt stress significantly influences the metabolic functions of both plant and rhizosphere bacteria, highlighting notable enhancements in specific metabolic pathways. Compared to CK, the treatments with SL and SH significantly enhanced the glycolysis/gluconeogenesis, arginine and proline metabolism, and pyruvate metabolism in plant bacteria (**Figure 5A**). As shown in **Figure 5C**, the bacterial secretion system and arginine and proline metabolism in rhizosphere bacteria were significantly increased in the SL, SZ, and SH treatment groups compared to CK. However, pyruvate metabolism was significantly decreased. To further understand the effects of different treatment levels on the metabolic functions of plant and rhizosphere bacteria, a one-way ANOVA was conducted on the functional attributes of plant bacteria across the different treatment groups. The results are presented in **Figures 5B, D**. In plant and rhizosphere bacteria, chemoheterotrophy and aerobic chemoheterotrophy exhibited distinct response patterns. Notably, significant expression of methanol oxidation and methylotrophy was observed in plant-associated bacteria. Nitrification activity in rhizosphere bacteria gradually increased under SL, SZ, and SH treatments.

### Confirming the potential role of Na^+^ and rhizobacterial flora in enhancing alfalfa adaptation to salt stress

**Figure 6:**
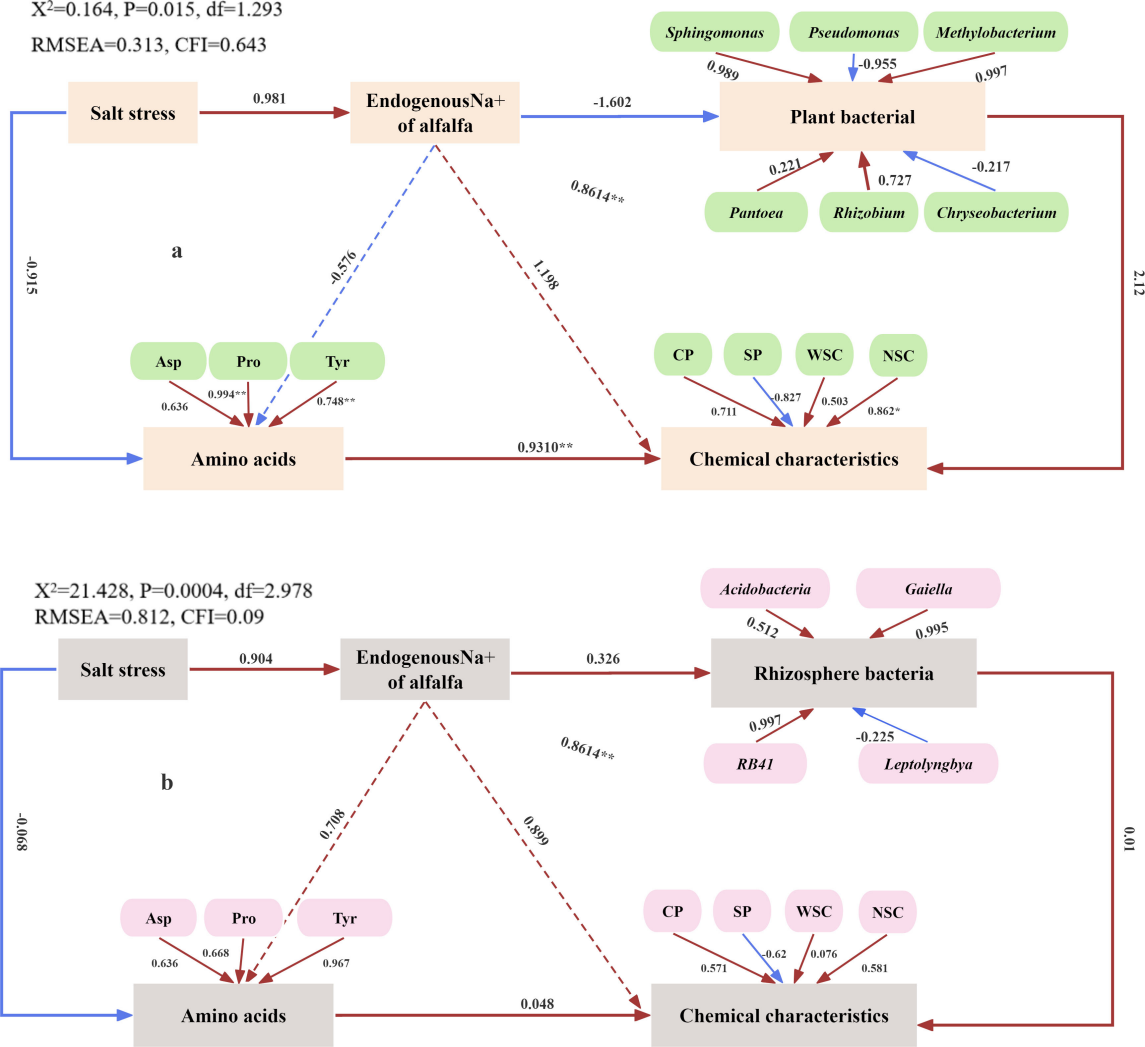
Potential regulation of plant **(A)** and rhizosphere **(B)** bacterial community and plant chemical composition by salt stress.

The SEM, an *a priori* algorithm, provides an intuitive representation that enhances understanding of the mechanisms driving the observed data. This model is used to investigate how Na^+^ affects plant and rhizosphere bacterial communities in enhancing plant adaptation to salt stress. **Figure 6A** shows a negative correlation between endogenous Na^+^ in alfalfa and plant bacteria (*R* = − 1.602) and a significant positive correlation with salt stress (*R* = 0.981). The loading coefficients of *Pseudomonas* and *Chryseobacterium* in plant bacteria are − 0.955 and − 0.217, respectively. These results indicate that the abundance of plant bacteria decreases with increasing salt stress, while the abundance of *Pseudomonas* and *Chryseobacterium* increases. A highly significant positive correlation was observed between amino acids and chemical characteristics (*R* = 0.931, *p* < 0.01), while a negative correlation with salt stress was also found (*R* = − 0.915). This suggests that as salt stress increases, both amino acid levels and chemical characteristics decline, negatively impacting the nutritional quality of alfalfa. Additionally, **Figure 6B** illustrates a positive correlation between the endogenous Na^+^ concentration in alfalfa and rhizosphere bacterial abundance (*R* = 0.326), as well as with salt stress (*R* = 0.904). The loading coefficient of *Leptolyngbya* in rhizosphere bacteria is − 0.225. These findings indicate that rhizosphere bacterial abundance increases with increasing salt stress, while *Leptolyngbya* abundance decreases. A positive correlation exists between amino acids and chemical characteristics (*R* = 0.048), while a negative correlation with salt stress (*R* = − 0.068) is observed. This suggests that increasing salt stress leads to decreased levels of amino acids and chemical characteristics, resulting in reduced nutritional quality.

**Figure 7:**
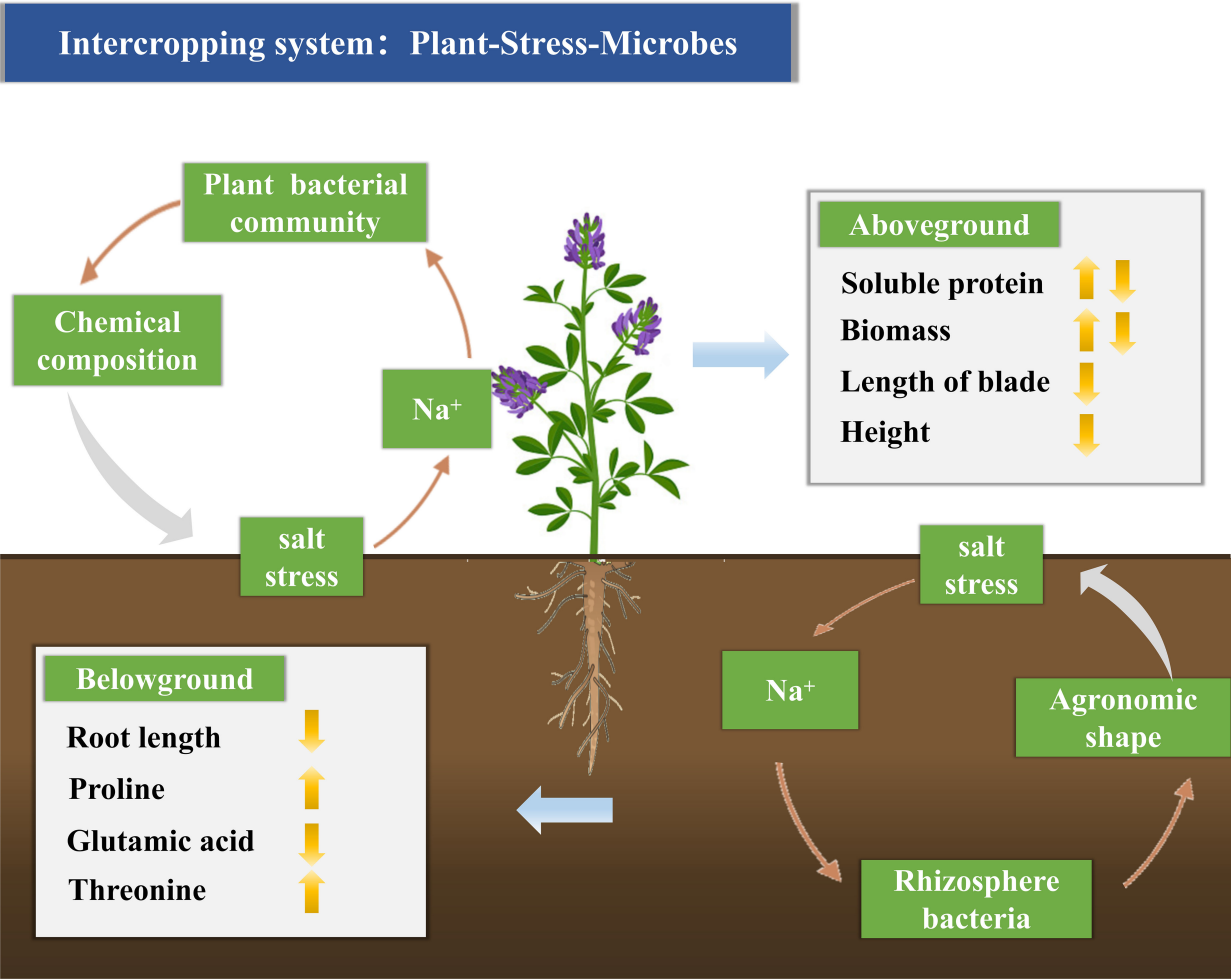
Conceptual model indicating the interaction between Na^+^ and plant and rhizosphere bacterial communities, significantly enhancing alfalfa’s ability to withstand salt stress. Na^+^ modifies the metabolism and community composition of plant and rhizosphere bacteria, thereby affecting the chemical characteristics of alfalfa. By adjusting the metabolism and community composition of plant and rhizosphere bacteria, Na^+^ can improve alfalfa’s ability to adapt to salt stress.

In summary, salt stress has distinct effects on plant bacterial and rhizosphere bacterial communities. As salt stress increases, plant bacterial abundance decreases, while rhizosphere bacterial abundance increases. Salt stress impairs alfalfa growth by altering its chemical composition (**Figure 7**).

## Discussion

### Effect of salt stress on chemical composition and soluble substances in alfalfa

Salt stress is a detrimental abiotic stressor that affects plant growth by impairing turgor, photosynthesis, and enzyme activity (Dzinyela et al., 2023). Bacteria can alleviate salt stress in plants by providing minerals and hormones while reducing ethylene production (Gao et al., 2022). This study investigated the impact of salt stress on alfalfa’s phenotypic characteristics, offering novel insights into alfalfa’s response to varying levels of salt stress. Salt treatments SH and SZ suppressed alfalfa growth and productivity (**Figure 1**). These findings align with previous studies demonstrating that high salt concentrations impact alfalfa growth. Middle NaCl salt stress reduced alfalfa plant height, leaf mass, and stem mass (Guo et al., 2024). During growth and development, excessive accumulation of Na^+^ hinders the uptake of water and nutrients, forcing plants to expend more energy on water and mineral absorption to maintain water potential and osmotic balance. This results in reduced growth rates and productivity (Gong, 2021; Mei et al., 2023).

In our study, SH treatment suppressed alfalfa’s underground biomass and root length, while SL treatment significantly increased underground biomass (**Figure 1**). These findings contrast with Zhao et al. (2021), who observed a reduction in aboveground biomass and root length under high-salinity conditions. This discrepancy may be attributed to a coordinated adaptive response in which biomass allocation between belowground and aboveground parts is adjusted, potentially accompanied by changes in nutrient utilization.

The modulation of various processes by proteins is essential for enhancing plant salt tolerance. These processes include photosynthesis, energy metabolism, ion homeostasis, gene transcription, protein biosynthesis, production of compatible solutes, and hormone regulation. These proteins may be employed in biotechnological strategies to enhance plant resilience in salt stress conditions. Soil salt stress is a major abiotic factor that limits plant growth and development, influencing the nutritional value of alfalfa (Li et al., 2022). Peng et al. (2008) showed that salt stress can increase the crude protein content of alfalfa within a specific range. Lin et al. (2018) observed an increasing trend in soluble sugars and soluble proteins in tomatoes under salt stress. In our study, the DM and CP content under different treatments initially increased and then decreased. Alfalfa treated with SZ had the highest crude protein content, which contrasts with the findings of previous studies. This discrepancy may be attributed to variations in salt tolerance among different plant species. Salt stress induces physiological drought in alfalfa, resulting in decreased water potential, altered stomatal regulation, reduced photosynthetic rate, and, ultimately, inhibited growth and nutrient accumulation (Greenway, 1980). The synthesis of small molecules in plants helps regulate osmotic pressure and enhances salt stress tolerance (Dzinyela et al., 2023). Cai et al. (2021) observed an increase in SOD protein abundance in honeysuckle under salt stress, which is consistent with the findings of this study.

The primary energy storage form in plants is NSC, which include soluble carbohydrates (e.g., sucrose and fructose) and starch, essential energy sources for plant growth and metabolism. WSC, mainly cellulose and lignin, are components of plant cell walls and structural tissues. The content of NSC and WSC in alfalfa follows a similar pattern to CP and DM, consistent with the findings of Morais et al. (2019). Salt stress alters the nutrient content in alfalfa, promoting the accumulation of crude protein and carbohydrates at appropriate levels of stress. Specific amino acids play protective roles in plants under stress conditions (Bertrand et al., 2016). Under different salt treatments, the contents of Thr and Pro increased, while the content of Glu decreased. The gradual increase in Na^+^ concentration under different treatments led to ion toxicity (**Figure 2B**) and triggered physiological and biochemical responses, resulting in the accumulation of ROS and secondary stress. The increase in Pro content helps plants by serving as an osmotic regulator and stabilizing cell membranes and proteins, thereby mitigating salt stress in alfalfa (Bertrand et al., 2016; Han et al., 2023). Previous studies have demonstrated that salt stress significantly increases proline accumulation (Han et al., 2023). The observed decrease in Glu content likely reflects its consumption or redistribution in response to stress, suggesting its role in maintaining cellular homeostasis under salt conditions.

### Salt stress-mediated effects on bacterial community structure in alfalfa

Salt stress poses a significant threat to plant growth and development. The rhizosphere microbial community plays a crucial role in plant adaptation to saline–alkaline environments (Cao et al., 2024). Microorganisms interact with plants to promote plant growth, both directly and indirectly, aiding in adaptation to adverse conditions. Growth-promoting bacteria secrete plant hormones such as auxins and indole-3-acetic acid, which aid in cell division and elongation (Forlani et al., 2019). Studies have demonstrated that plant growth-promoting bacteria can substantially enhance plant growth. Timmusk et al. (2011). Research has shown that plants without microbial symbiosis are more susceptible to diseases and have lower survival rates in natural environments. Bacterial community composition varies across plants, influenced by environmental factors such as soil structure, pH, fertility, drought, and salinity (Laiq et al., 2024). This study investigated changes in the richness of alfalfa bacterial communities under different treatment levels. The predominant phyla in the alfalfa bacterial community are *Proteobacteria*, *Actinobacteria*, *Bacteroidetes*, and *Firmicutes*. Notably, salinity stress influenced the abundance of *Bacteroidetes* and *Proteobacteria*. The abundance of *Proteobacteria* initially increased, likely due to their role in promoting plant growth, but later decreased as some bacteria reached their tolerance limits, and salt ions inhibited microbial growth and reproduction. This trend aligns with the findings of Wang et al. (2021).

The abundance of *Bacteroidetes* increased across the four treatments, suggesting its adaptation to environments with higher salt content. Under saline conditions, significant changes were observed in certain genera, such as *Pantoea*, *Pseudomonas*, *Chryseobacterium*, and *Flavobacterium*, which play crucial roles in plant growth and development. Existing literature highlights the ability of these genera, particularly *Pseudomonas*, to maintain ion balance or homeostasis in plant roots under high salt conditions. This not only helps mitigate the toxic effects of salt but also promotes plant growth, reducing crop losses caused by salinity (Singh et al., 2020). *Flavobacterium* has been found to enhance plant tolerance to salt stress. For instance, Kim et al. (2020) used *Flavobacterium* HYN0056(T) to improve the salt stress tolerance of *Arabidopsis*. This could also contribute to the increased CP of alfalfa in saline–alkali soil.

Under abiotic salt stress, symbiotic microorganisms, such as *Pseudomonas*, may contribute to improved plant growth, in addition to the plant’s intrinsic salt and ion stress tolerance mechanisms. *Pseudomonas* can alleviate the impact of salt stress on plants (Berni et al., 2022). The increased abundance of *Pseudomonas* aids alfalfa growth and development in saline environments, consistent with the findings of Egamberdieva et al. (2013). The rhizosphere is home to abundant and diverse microbial communities (Lemanceau et al., 2017). Previous studies have highlighted the role of rhizosphere microbial communities in promoting plant adaptation to salt stress (Bai et al., 2023). Under different treatment levels, the abundance of *Actinobacteria* and *Acidobacteria* increased significantly, suggesting their ability to promote plant growth under salt stress conditions (Rangseekaew et al., 2022). *Cyanobacteria* can regulate alfalfa growth under salt stress by promoting increased nutrient uptake. The decreasing abundance of *Cyanobacteria* across different treatments indicates their reduced tolerance to high salt stress conditions. *Leptolyngbya* contributes to energy metabolism and material cycling in alfalfa; however, its decreasing abundance under different treatments suggests that high salt stress inhibits *Leptolyngbya* growth, leading to reduced root and leaf lengths in alfalfa under the SH treatment.

The adaptive mechanisms of alfalfa and its associated bacteria to salt stress involve complex metabolic and functional changes.

In natural ecosystems, plants employ various mechanisms to adapt to both biotic and abiotic stress. These mechanisms include regulating glutathione metabolism, producing secondary metabolites, and synthesizing compounds such as sesquiterpenes, triterpenes, and carotenoids. Additionally, plants may break down limonene or pinene, metabolize sugars and glycerides, and produce methyl butyrate to cope with stress conditions (Handayani et al., 2019; Ghatak et al., 2023). **Figure 1** explores the effects of salt stress on the amino acid content of alfalfa, specifically examining changes in Thr, Glu, and Pro levels. To better adapt to the saline environment, the functions and metabolism of plant-associated bacteria and rhizosphere bacteria in alfalfa underwent adjustments. Significant expressions of methanol oxidation and methylotrophy are observed in **Figure 5B**, possibly due to enhanced bacterial functions promoting glycolysis/gluconeogenesis, arginine and proline metabolism, and pyruvate metabolism in plant-associated bacteria, which help produce necessary amino acids for survival under salt stress (Har et al., 2021). This finding is consistent with the results of Damaris et al. (2016) in their proteomic study of salt stress responses in rice seedlings. High Na^+^ concentrations profoundly affected the metabolic functions of rhizosphere bacteria. To adapt to the saline environment, rhizosphere bacteria enhanced aerobic chemoheterotrophy and chemoheterotrophy, thereby enhancing the bacterial secretion system and arginine and proline metabolism. This result is similar to the findings of Luo et al. (2024). The decrease in pyruvate metabolism may result from excessive Na^+^ in the soil, affecting the assembly and function of rhizosphere bacteria (Yu et al., 2021).

## Conclusion

Our research indicates that the enhanced adaptability of alfalfa to salt stress is primarily attributed to the interactions between Na^+^ and plant and rhizosphere bacterial communities. The data highlight differences in the metabolism and community composition of plant and rhizosphere bacteria, emphasizing the link between bacterial metabolism and community composition, and the chemical characteristics of alfalfa. Na^+^ has a significant impact on the adaptability of alfalfa to salt stress by modulating the metabolism and community composition of plant and rhizosphere bacteria. Our experiments further demonstrate that Na^+^ indirectly affects alfalfa’s adaptability to salt stress by influencing the metabolism and community composition of plant and rhizosphere bacteria. These findings emphasize the significance of Na^+^ interactions with plant and rhizosphere bacterial communities in enhancing alfalfa’s adaptability to salt stress. They also provide valuable insights for future production practices, highlighting the potential to enhance plant performance by harnessing specific microbial groups.

## Data Availability

The datasets presented in this study can be found in online repositories. The names of the repository/repositories and accession number(s) can be found in the article/**Supplementary Material**.
