# Supplementary material for: Effects of salt stress on plant and rhizosphere bacterial communities, interaction patterns, and functions
Source: Front Plant Sci. 2025 Jan 9;15:1516336. doi: 10.3389/fpls.2024.1516336 (PMC11753915; doi:10.3389/fpls.2024.1516336)
Supplement: Supplementary file 1 [file Table1.docx]

| **Supplement table 1.**  The physical and chemical properties of soils. | | | | |  |  |
| --- | --- | --- | --- | --- | --- | --- |
| Indictors | Na^+^（g/kg） | K^+^（g/kg） | pH | EC (mS/cm) | SO_4_^2-^（g/kg） | Cl^-^（g/kg） |
| CK | 0.11± 0.006c | 0.027±0.001d | 7.4±0.21b | 0.2±0.02a | 0.008±0.01b | 0.051±0.02c |
| SL | 0.15±0.004b | 0.031±0.001c | 8.4±0.08a | 0.6±0.10b | 0.023±0.01a | 0.119±0.01b |
| SZ | 0.16±0.0020b | 0.035±0.001b | 8.6±0.11a | 1.3±0.01c | 0.024±0.01a | 0.125±0.01b |
| SH | 0.25±0.019a | 0.041±0.001a | 8.7±0.34a | 2.3±0.29d | 0.027±0.01a | 0.203±0.02a |
| Numbers in a column followed by different lowercase letters differ at *P* < 0.05. EC, electrical conductivity. | | | | |  |  |
